# Account of Diseases in an Eastern District of London, from the 20th of August, to the 20th of September, 1801

**Published:** 1801-10

**Authors:** 


*Account of Diseases in an Eastern District of London, from the 20th of August, to the 20th of September,* 1801.

| | |
|---|---|
| ACUTE DISEASES. | |
| Typhus | 25 |
| Febris Remittens | 1 |
| Variola | 1 |
| Peripneumonia | 2 |
| Acute Rheumatifm | 3 |
| Dyfenteria | 10 |
| CHRONIC DISEASES. | |
| Tuffis | 8 |
| Tuffis et Dyfpnœa | 6 |
| Peripneumonia Notha | 2 |
| Phthifis Pulmonalis | 3 |
| Hepatitis Chronica | 3 |
| Anafarca | 7 |
| Gaftrodynia | 3 |
| Dyfpepfia | 4 |
| Tenefmus | 1 |
| Diarrhœa | 6 |
| Enterodynia | 4 |
| Hæmorrhois | 3 |
| Menorrhagia | 4 |
| Fluor Albus | 2 |
| Scrophula | 3 |
| Vomitus | 6 |
| Vertigo | 3 |
| Paralyfis | 2 |
| Impetigo | 3 |
| Lumbago | 2 |
| Rheumatifmus Chronicus | 12 |
| PUERPERAL DISEASES. | |
| Menorrhagia Lochialis | 4 |
| Dolores Poft Partum | 3 |
| INFANTILE DISEASES. | |
| Diarrhœa | 6 |
| Ophthalmia | 2 |
| Aphthæ | 3 |

As the feafon of the year has arrived in which fevers of the Typhus kind ufually conftitute a large part of the catalogue of difeafes, the number in the prefent lift will not excite any furprife. The principal occafion of regret is, that during thofe months in which, in the common courfe of things, this clafs of difeafes does not make a confpicuous figure, it has lately very much engaged the attention of practitioners. In our laft Report we were happy to announce, that the difeafe had affumed a milder form, that the affections of the brain were lefs violent, and that the inftances of a fatal termination were lefs frequent. In the cafes which have fince occurred, the delirium has been lefs violent; it has been rather of that low muttering kind which we have been formerly accuftomed to obferve in this fpecies of fever. But though the brain has been lefs violently affected, the effects of the difeafe upon this organ have continued longer than ufual.

In one inftance, after the termination of the fever, the patient fell into that ftate of intellectual derangement which Nofologifts have diftinguifhed by the term Amentia.

A general inattention to furrounding objects, and an indifference to every perfon and to every thing about them, together with an indifpofition to take in neceffary food, are characteriftics of this difeafe. The patient referred to, continued in this ftate for feveral days, but feems now to be gradually recovering from it. In one of the cafes, fymptoms of Hemiplegia occurred at the clofe of the fever ; and in another, fuch a lofs of recollection and other fymptoms of mental debility as produced the appearance of premature old age.

Dyfenteries have very frequently occurred during the laft few weeks. Thefe have been attended with the ufual fymptoms of a mild difeafe, and have not, in any of the cafes referred to, proved fatal.

